# Australian Community Pharmacists’ Experience of Implementing a Chronic Kidney Disease Risk Assessment Service

**DOI:** 10.5888/pcd15.170485

**Published:** 2018-06-14

**Authors:** Pankti A. Gheewala, Gregory M. Peterson, Syed Tabish R. Zaidi, Matthew D. Jose, Ronald L. Castelino

**Affiliations:** 1 Division of Pharmacy, School of Medicine, Faculty of Health, University of Tasmania, Hobart, Australia; 2 School of Medicine, Faculty of Health, University of Tasmania, Hobart, Australia; 3 Sydney Nursing School, The University of Sydney, Sydney, Australia

## Abstract

**Introduction:**

Community pharmacists are well positioned to deliver chronic kidney disease (CKD) screening services. However, little is known about the challenges faced by pharmacists during service implementation. This study aimed to explore community pharmacists’ experiences and perceived barriers of implementing a CKD risk assessment service.

**Methods:**

Data collection was performed by using semistructured, open-ended interview questions. Pharmacists who had implemented a CKD screening service in Tasmania, Australia, were eligible to participate. A purposeful sampling strategy was used to select pharmacists, with variation in demographics and pharmacy location. A conventional content analysis approach was used to conduct the qualitative study. Transcripts were thematically analyzed by using the NVivo 11 software program. Initially, a list of free nodes was generated and data were coded exhaustively into relevant nodes. These nodes were then regrouped to form highly conceptualized themes.

**Results:**

Five broad themes emerged from the analysis: contextual fit within community pharmacy; perceived scope of pharmacy practice; customer perception toward disease prevention; CKD – an underestimated disease; and remuneration for a beneficial service. Pharmacists found the CKD service efficient, user-friendly, and of substantial benefit to their customers. However, several pharmacists observed that their customers lacked interest in disease prevention, and had limited understanding of CKD. More importantly, pharmacists perceived the scope of pharmacy practice to depend substantially on interprofessional collaboration between pharmacists and general practitioners, and customer acknowledgment of pharmacists’ role in disease prevention.

**Conclusion:**

Community pharmacists perceived the CKD service to be worth incorporating into pharmacy practice. To increase uptake, future CKD services should aim to improve customer awareness about CKD before providing risk assessment. Further research investigating strategies to enhance general practitioner involvement in pharmacist-initiated disease prevention services is also needed.

**MEDSCAPE CME**

Medscape, LLC is pleased to provide online continuing medical education (CME) for this journal article, allowing clinicians the opportunity to earn CME credit.

In support of improving patient care, this activity has been planned and implemented by Medscape, LLC and Preventing Chronic Disease. Medscape, LLC is jointly accredited by the Accreditation Council for Continuing Medical Education (ACCME), the Accreditation Council for Pharmacy Education (ACPE), and the American Nurses Credentialing Center (ANCC), to provide continuing education for the healthcare team.

Medscape, LLC designates this Journal-based CME activity for a maximum of 1.00 *AMA PRA Category 1 Credit(s)™*. Physicians should claim only the credit commensurate with the extent of their participation in the activity.

All other clinicians completing this activity will be issued a certificate of participation. To participate in this journal CME activity: (1) review the learning objectives and author disclosures; (2) study the education content; (3) take the post-test with a 75% minimum passing score and complete the evaluation at http://www.medscape.org/journal/pcd; (4) view/print certificate.

Release date: June 14, 2018; Expiration date: June 14, 2019

**Learning Objectives**

Upon completion of this activity, participants will be able to:

1. Assess trends in the epidemiology and management of chronic kidney disease (CKD)
2. Analyze pharmacists' view of the contextual fit of CKD risk assessment in their practice
3. Evaluate pharmacists' response regarding CKD risk assessment in the scope of their practice
4. Distinguish barriers to a successful program of CKD risk assessment in pharmacies

**EDITOR**

Kate W. Harris

Editor, *Preventing Chronic Disease*

Disclosure: Kate W. Harris has disclosed no relevant financial relationships.

**CME AUTHOR**

Charles P. Vega, MD, FAAFP

Health Sciences Clinical Professor, University of California, Irvine, Department of Family Medicine; Associate Dean for Diversity and Inclusion, University of California, Irvine, School of Medicine; Executive Director, University of California, Irvine, Program in Medical Education for the Latino Community, Irvine, California

Disclosure: Charles P. Vega, MD, FAAFP, has disclosed the following relevant financial relationships:

Served as an advisor or consultant for: Johnson and Johnson Healthcare

Served as a speaker or a member of a speakers bureau for: Shire Pharmaceuticals

**AUTHORS**

Pankti A. Gheewala, BPharm (Honours)

Division of Pharmacy, School of Medicine, Faculty of Health, University of Tasmania

Disclosure: Pankti A. Gheewala, BPharm (Honours), has disclosed no relevant financial relationships.

Gregory M. Peterson, BPharm (Honours), PhD

Division of Pharmacy, School of Medicine, Faculty of Health, University of Tasmania

Disclosure: Gregory M. Peterson, BPharm (Honours), PhD, has disclosed no relevant financial relationships.

Syed Tabish R. Zaidi, MPharm, PhD

Division of Pharmacy, School of Medicine, Faculty of Health, University of Tasmania

Disclosure: Syed Tabish R. Zaidi, MPharm, PhD, has disclosed no relevant financial relationships.

Matthew D. Jose, MBBS, PhD

School of Medicine, Faculty of Health, University of Tasmania

Disclosure: Matthew D. Jose, MBBS, PhD, has disclosed no relevant financial relationships.

Ronald L. Castelino, MPharm, PhD

Sydney Nursing School, The University of Sydney, Australia

Disclosure: Ronald L. Castelino, MPharm, PhD, has disclosed no relevant financial relationships.

## Introduction

Globally, more than 497 million adults (aged ≥20 years) had chronic kidney disease (CKD) in 2010 (1). In Australia, at the end of 2015, the number of patients receiving renal replacement therapies (such as dialysis or transplantation) was 968 per million population and rising (2). CKD causes a huge economic burden for the health care system, and the cost to the Australian government for treating end-stage kidney disease (ESKD), between 2009 and 2020, is estimated to be between $11.3 billion and $12 billion AUD (3).

CKD is common among older Australian adults in primary care settings; a 2012 study reported abnormal kidney function in 37% of primary care patients (4). Evidence suggests that early diagnosis and management of CKD can reduce the risks of disease progression and associated cardiovascular disease (CVD) by as much as 50% (5). Despite this, CKD is often under-recognized, and in 2015 in Australia, 17% of new patients received late referrals to nephrologists for management of ESKD (2). Additionally, testing for CKD in Tasmania was suboptimal; serum creatinine measurement was performed during 12 months for only 50.6% of at-risk individuals and albuminuria was measured in only 9.4% of people with an estimated glomerular filtration rate (eGFR) of less than 60 mL/min per 1.73 m^2^ (6). This indicates a large gap in clinical practice and the need to explore ways to improve early detection of CKD.

Worldwide, several clinical practice guidelines recommend screening targeted groups of people with established risk factors as an important strategy for early CKD detection (7). Hence, many community-based targeted CKD screening programs have been conducted globally; however, a systematic review evaluating the effectiveness of these programs reported that most interventions lacked methodologic rigor and long-term feasibility (8). Instead, Kidney Health Australia (KHA) identified implementation of targeted “opportunistic” CKD screening within the Australian health care system as a more feasible approach (9,10). Opportunistic screening occurs when a check or test is offered to a person with no CKD symptoms who has come to the health care system for other reasons (10).

Currently, in Australia, routine screening for CKD is not practiced (10). Involving community pharmacists in early CKD detection could prove beneficial (11). In recent years, the role of pharmacists has extended to providing disease prevention services (12,13), and pharmacy screening for conditions such as diabetes (14), osteoporosis (15), CVD (16), and atrial fibrillation (17) has shown potential. Similarly, a community pharmacist–initiated targeted CKD risk assessment service could help to alert general practitioners (GPs) of at-risk patients who need further diagnostic evaluation.

Current literature indicates that risk assessment tools can facilitate early identification of people at risk of developing CKD. One such validated tool recommended by KHA is the QKidney risk calculator (18,19), which estimates a person’s 5-year risk of developing moderate to severe CKD. After a CKD risk assessment service was developed using this tool, all Tasmanian community pharmacies were invited to participate in the CKD risk assessment study. Consequently, the CKD service was implemented and evaluated at 24 pharmacies (20). Because this was a pilot program, determining the feasibility of the CKD service and making refinements were necessary before widespread adoption in the community pharmacy setting. Then a follow-up qualitative study was conducted. The aim of this study was to explore pharmacists’ experiences of implementing the CKD service, with a specific objective of identifying perceived barriers to service implementation.

## Methods

### Ethical approval

The Tasmanian Social Sciences Human Research Ethics Committee (H0015669) approved this study.

### CKD risk assessment service

Before implementing the CKD service, online training was provided for participating pharmacists, and their skills and knowledge associated with CKD risk assessment were evaluated (21). Customer participation for CKD risk assessment was promoted by displaying a poster in each participating pharmacy; pharmacists also approached eligible customers directly in the pharmacy. Customer eligibility criteria for participation is described in the CKD risk assessment protocol as shown in the Figure. The online QKidney risk calculator (18,19) was used to identify participants with ≥3% risk; these people were counselled on the results, given educational materials, and advised to consult their GP for further assessment. After 9 months, participants were followed up and their laboratory data collected from a pathology provider to determine the percentage of participants who underwent eGFR and urine albumin creatinine ratio (ACR) measurement. Follow-up data analysis showed relatively low GP referral uptake (27%), and pathology analysis revealed suboptimal kidney testing in 80% of participants with ≥3% risk.

**Figure:**
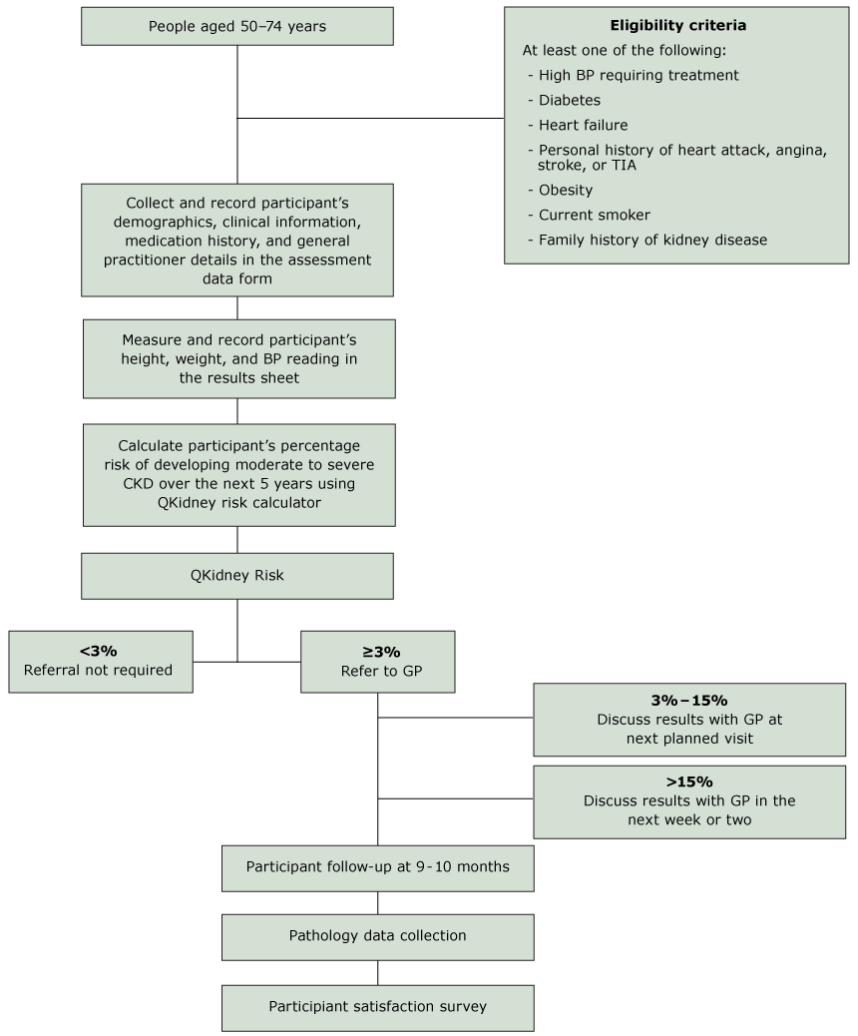
Flow diagram for chronic kidney disease risk assessment protocol. Abbreviations: BP, blood pressure; CKD, chronic kidney disease; GP, general practitioner; TIA, transient ischemic attack.

### Study design and participant selection

Initially, researchers explained the purpose and design of the study to the pharmacists by mail and/or telephone. Written informed consent was obtained from all 24 pharmacists before service implementation. After the intervention, P.G. telephoned 8 pharmacists to organize the face-to-face qualitative interview. Participants were selected using a purposeful sampling strategy, to provide variation in demographics and pharmacy location (22). No pharmacists refused to participate in the qualitative interview.

### Data collection

A semistructured, in-depth interview was conducted with individual pharmacists at their respective community pharmacies, 4 months after completion of the intervention. Interview questions were open-ended and directly related to the study objectives (Box). Prompts were also offered to the pharmacists when open-ended questions elicited little response. All interviews were audio-recorded and were 15 to 45 minutes long.

**Box. Interview Guide for Pharmacists**

**Table:** 

Would you please describe your daily role as a community pharmacist? Could you please tell me the demographics of most of the customers that present to your pharmacy? Do you have a doctor’s surgery nearby?
What do you think is the current role of community pharmacists in providing health promotion, risk assessment and screening services?
What did you think about the chronic kidney disease risk assessment study? Follow-up questions: -What did you like most about this service? -Was there any aspect of this service that you didn’t like? -Was there any aspect you thought could be improved?
According to you, is the QKidney risk assessment calculator feasible or useful in a community pharmacy setting?
What was it like to recruit participants for this study? Follow-up questions: -What strategy did you use to recruit participants? -How did you find the recruitment process? -Did you find it difficult or easy to recruit participants? -Could you give me examples of a few reasons that participants used for declining to participate in this study?
How did your participants respond to this risk assessment service? Did they seem interested? Did your participants know what chronic kidney disease is before participation? Follow-up questions: -What did your patients think about it? -What was their reaction? -Did they come back asking about it? -Have you received any feedback from participants?
Did you have your own target in mind that you would recruit at least X number of participants for this study and were you able to accomplish your target? Follow-up questions: -What prevented you from achieving your target?
What do you think stops people in the community from participating in a pharmacist-led chronic kidney disease risk assessment service?
Do you think people in the community need a chronic kidney disease risk assessment service?
Have you had any feedback from a general practitioner or a nurse with regards to the chronic kidney disease risk assessment service?
Overall, would you consider the chronic kidney disease risk assessment service to be a success? Why? Follow-up question: -I am curious to know whether you would be willing to offer this service in your community pharmacy if you had a choice.
Finally, if there was anything that you would have changed or done differently when it comes to implementing similar research projects in your community pharmacy in future, what or how would you do it? Thank you once again for participating in this study.

### Data analysis

QSR International’s NVivo 11 software program was used to support each phase of qualitative data analysis. All audio recordings were transcribed verbatim into Microsoft Word documents by Digitype Australasia (a specialized legal and medico-legal transcription service) or P.G., and imported into NVivo. A conventional approach for content analysis was used to perform the qualitative research (23). In this type of analysis, the use of preconceived categories or theoretical perspectives is avoided, and coding categories are derived directly from the data (participants’ unique perspectives). Hence, this approach was deemed most appropriate and used to gain further insight into the pharmacists’ experiences of implementing the CKD service.

The general inductive approach (24) was used to identify themes in the documents that were relevant to study objectives. Initially, a list of free nodes (data categories) was generated by studying the transcripts repeatedly, and document contents were thoroughly coded into relevant nodes. These nodes were then regrouped and organized hierarchically into trees to establish highly conceptualized themes. Initial coding of the raw data was performed by P.G., with a subsequent discussion with a second author (S.T.Z.) to reach an agreement on the themes. These themes were then applied back to the original data. The research team met again to further refine the themes, which were reviewed against the original transcripts. Feedback from participants on results of the analysis was not obtained. The consolidated criteria for reporting qualitative research (COREQ) were followed (25).

## Results

Eight pharmacists were interviewed and data saturation was met. The Table summarizes pharmacist characteristics. Five major themes emerged from the analysis: contextual fit within community pharmacy; perceived scope of pharmacy practice; customer perception toward disease prevention; CKD – an underestimated disease; and remuneration for a beneficial service.

**Table:** Characteristics of Interviewed Pharmacists

Pharmacist’s No.	Age Group (y)	Gender	Years Worked as a Community Pharmacist	Pharmacy Location^a^	No. of Participants Recruited
P1	56–65	Male	11–15	S	>30
P2	36–45	Male	5–10	S	10–20
P3	56–65	Female	>20	N/NE	<10
P4	26–35	Female	<5	N/NE	>30
P5	36–45	Male	5–10	S	<10
P6	26–35	Female	<5	S	<10
P7	46–55	Female	16–20	NW/WW	<10
P8	46–55	Male	>20	NW/WW	<10

Abbreviations: N/NE north/northeast; NW/WW northwest/western wilderness; S south.

a Location of the pharmacy in the state of Tasmania is described geographically.

### Contextual fit within community pharmacy

This theme was defined as the appropriateness of providing the CKD risk assessment service within the context of community pharmacy practice, as perceived by the pharmacists. Most pharmacists in this study agreed that the CKD risk assessment was a well-designed and straightforward service, which could be incorporated into their routine practice.

I thought that it was a good idea as a screening. You know it was nice and simple to go through with the patients. It didn’t take too long. (P5)

Additionally, pharmacists identified the online risk assessment calculator as a user-friendly tool. They liked the online calculator’s minimum data requirement, which could be easily obtained from participants and provided results immediately.

I think it’s definitely a good tool to have and it doesn’t exactly take very long, so it’s not like it would take that much time out of your day to do a quick check. (P4)

Most pharmacists mentioned that customers who underwent risk assessment appreciated the service.

Any of them that clipped up (were identified with ≥3% risk), they were in the doctors straightaway…and they thought that was good…it was just really positively received by all the participants. (P3)

However, several pharmacists mentioned that, given the nature of a community pharmacy business, it was sometimes challenging to recruit participants and carry out risk assessment. Being the sole pharmacist on duty or having a busy pharmacy prohibited pharmacists from performing risk assessment.

Our time was little bit tricky to squeeze it in. Trying to come out and approach patients about this service and that was a little bit of a barrier I guess than trying to fit it between prescriptions. (P2)

Some pharmacists reported that the paperwork required too much time and thinking, and further suggested streamlining or computerizing the entire process.

I think it could be quite easily streamlined so that it becomes easy to do but it does need to be a real, simple process for the pharmacist….(P3)

Several pharmacists found that training staff members to identify eligible customers, and to recruit and perform the initial stages of the risk assessment, was a useful way to reduce their burden.

As soon as we started teaching the staff members, you know make sure that you ask them this, then it became a lot easier. (P4)

### Perceived scope of pharmacy practice

As perceived by pharmacists, the continuing scope of pharmacy practice (especially in providing disease prevention services) was found to be highly dependent on the interprofessional collaboration between pharmacists and GPs, and on customers’ acknowledgment of the role of pharmacists in disease prevention. Pharmacists reported difficulty in engaging GPs as a key barrier to the perceived effectiveness of the CKD service. At the beginning of the study, several pharmacists and GPs had a brief discussion on the CKD service. Pharmacists received mixed reactions: some GPs had a positive view of risk assessment while others did not actively participate. However, most pharmacists said that they did not receive any feedback from the GPs, and some wanted to know the outcome of GP referral. Pharmacists expressed that, although they can provide such services, having GPs on board was crucial. GPs were considered the final decision makers in the care of patients; without their support, pharmacists believed that the scope of the CKD service was limited.

We did communicate that [CKD service] to them [GPs] briefly but you know they’re very busy. They didn’t take it on board as a big deal. (P2)

Several pharmacists felt that their professional standing in disease prevention was still undervalued, with their customers generally not perceiving this as a role for pharmacists.

I think this is an area still to be developed. I am not sure that in a general community our credentials as screening people have been developed or promoted…to the point for people [to] actually see us as being preventative provider. (P8)

However, integration of the CKD service with other established pharmacy services, for which pharmacists have credibility (eg, medication review and diabetes management), was found to improve service acceptability and uptake.

What I did is your kidney study was there and then diabetes study we started in the pharmacy, so we linked both together and that has been better. So the same person, we can sometimes do both studies. (P1)

To encourage customer participation, some pharmacists used flyers or newsletters (in addition to the promotional poster), and invested more time in directly approaching and explaining the service to eligible customers.

We went okay once we started going out and really putting posters up and that sort of thing. (P4)

Several pharmacists stated that large-scale promotion through the media could help to create awareness and improve the uptake of pharmacy services.

It’s probably the fact that we don’t have ads on the radio saying go into pharmacy for this and the other…that’s the thing that makes people realize what your scope of business is. (P7)

### Customer perception of disease prevention

Pharmacists stated that customers were often unwilling to participate, mainly due to their lack of interest in disease prevention.

The people who I thought would be good to do really didn’t – they weren’t interested. They didn’t have time for their health basically. They weren’t worried enough about it. (P3)

Pharmacists also found it difficult to engage customers for participation.

A lot of the time they [customers] will come in and they are busy…. So they will be like aw I have only got enough time to get my scripts while I am here. Even though someone could be doing it while they were waiting for the script. But they don’t seem to see to both of them together. They just go nope can’t do it, I don’t have enough time…. (P6)

### CKD – an underestimated disease

Pharmacists frequently noted that limited CKD awareness campaigns might have contributed to the reduced uptake of the CKD service. Pharmacists perceived CKD to be an important, yet often ignored, chronic disease in the community. They mentioned that, compared with other diseases such as diabetes, hypertension, or CVD, CKD and its screening are not promoted on a large scale within the community.

I think diabetes screening is actually more prominent simply because of the fact that there is more funding behind it and Diabetes Tasmania tends to promote it reasonably well in terms of people getting screened. So again it’s probably an awareness thing I think that they have more awareness than kidney function possibly does. (P8)

Additionally, pharmacists found that their customers had a limited understanding of CKD and kidney functions. Because CKD is asymptomatic, customers believed that their kidneys were fine; hence, they did not see the need for risk assessment.

They didn’t think it was something you know they needed to look out or they felt fine at that particular moment, so why would they worry about what their kidneys were doing? (P7)

### Remuneration for a beneficial service

Pharmacists perceived the CKD service to be of substantial benefit to their customers. They found that the service helped to raise participants’ awareness about the kidneys and improved their understanding of CKD.

I think the questions [of CKD service] actually [helped] awaken people to the possibility that [kidneys] could actually be having influence on their life. A lot of people don’t necessarily realize that…they are likely to have or develop kidney problems…. I think knowing…the likelihood of that sort of outcome [CKD] is actually useful information once they [patients] have realized the importance of it. (P8)

Most pharmacists mentioned that they would like to be remunerated for providing the CKD service, especially if it were to become an essential component of the pharmacy business. One pharmacist stressed that if the service was funded, then they would be able to allocate more of their own and their staff’s time to it.

There had to be some sort of remuneration…so it makes it [CKD service] worth the time…. At the end of the day, we have to run a business and pay for staff so to be able to prioritize time for those different jobs you need to have some sort of income for it. (P7)

## Discussion

Overall, the community pharmacists believed that the CKD risk assessment service was worth incorporating into pharmacy practice. Pharmacists perceived that the service would be of benefit to their customers and important to raising CKD awareness in the community. However, during service implementation, pharmacists experienced several challenges; difficulty in engaging GPs was a crucial barrier to the long-term feasibility of the service. Although interprofessional collaboration between pharmacists and GPs is vital for complete patient care, overall GP engagement was minimal, even after initial discussion of the CKD service between some pharmacists and GPs. One reason could be the extent and mode of the discussion (26). In Australia, the role of community pharmacists in providing public health and disease prevention services is still developing. A qualitative study of Australian GPs showed that most did not favor pharmacists’ providing screening services, as they believed screening to be the role of the GP, and they lacked confidence in pharmacists’ clinical expertise and competence to perform screening services (26). Conversely, the study found that GPs were generally supportive of those pharmacy services that they found useful in managing patients, such as medication reviews.

Another barrier to service implementation was the limited customer perception of pharmacists’ scope of practice. A qualitative study exploring the awareness and views of Australian community pharmacy practice showed that most customers viewed pharmacists primarily as medication suppliers and had limited knowledge about pharmacy practice (27). Similar findings were reported by another study conducted in Scotland (28), which additionally reported that customers lacked confidence in pharmacists’ extended role. However, a systematic review examining customer attitudes found that they were more receptive to the availability of medicine-related services than health promotion or screening services, but those who experienced these pharmacy services were highly satisfied with them (12). In a recent Australian atrial fibrillation screening study, pharmacists perceived combining screening with other established services, such as medication reviews, as an alternative approach to improve service uptake (29). Similarly, in this qualitative study, pharmacists observed an improvement in the customers’ response to the CKD service when it was integrated with other professional services.

Previous studies have found that, to improve customer awareness and participation in pharmacy services, pharmacists need to reassess their daily practice and ensure that they are not just dispensing prescriptions (27,29). Instead, pharmacists should play an active role by directly approaching customers and explaining available services, and by using prominent flyers (29). Similarly, pharmacists in this study found investing more time in explaining the service to customers, and the use of additional flyers or newsletters, improved the service implementation process.

This study identified limited customer understanding of CKD and kidneys as a potential barrier to service uptake. A study exploring how the public decides to undergo health checks for CVD prevention found that the decision depends on their perception of being vulnerable to the disease and having any symptoms (30). Similarly, lack of symptoms was identified as a barrier to CKD service uptake. Kidney function can decline by as much as 90% before symptoms appear (10), and lack of this knowledge puts a person at increased risk when diagnosis is delayed. Hence, providing customers with information on CKD before offering risk assessment might improve their understanding and make them more receptive to the service.

Customers and GPs were not interviewed in this study. Exploring their perceptions of the CKD service may help to identify additional barriers, and addressing these could help the pharmacists enhance service implementation. Although only a few pharmacists were interviewed in this study, in studies with a relatively homogenous sample population and narrow objectives, interviews with as few as 6 subjects are sufficient to reach saturation, with meaningful themes and valuable interpretation (31).

Overall, pharmacists perceived implementation of the CKD risk assessment service to be feasible in community pharmacy practice. However, because of the importance of GPs in treating CKD, pharmacists need to demonstrate their professional expertise to GPs, explain the benefits of the CKD service, and establish mutual understanding on the referral method. Next, during training, pharmacists should be made aware of the strategy of integrating the CKD service with other established services commonly used by their pharmacy customers. This has the potential to improve customer participation and increase service uptake. Also, pharmacists should play an active role in promoting and delivering the CKD service and aim to improve customer awareness about CKD before offering risk assessment. These strategies may help to improve the feasibility of the CKD risk assessment service. However, future research evaluating these strategies is needed.

## Post-Test Information

To obtain credit, you should first read the journal article. After reading the article, you should be able to answer the following, related, multiple-choice questions. To complete the questions (with a minimum 75% passing score) and earn continuing medical education (CME) credit, please go to http://www.medscape.org/journal/pcd. Credit cannot be obtained for tests completed on paper, although you may use the worksheet below to keep a record of your answers.

You must be a registered user on http://www.medscape.org. If you are not registered on http://www.medscape.org, please click on the “Register” link on the right hand side of the website.

Only one answer is correct for each question. Once you successfully answer all post-test questions, you will be able to view and/or print your certificate. For questions regarding this activity, contact the accredited provider, CME@medscape.net. For technical assistance, contact CME@medscape.net. American Medical Association’s Physician’s Recognition Award (AMA PRA) credits are accepted in the US as evidence of participation in CME activities. For further information on this award, please go to https://www.ama-assn.org. The AMA has determined that physicians not licensed in the US who participate in this CME activity are eligible for *AMA PRA Category 1 Credits™*. Through agreements that the AMA has made with agencies in some countries, AMA PRA credit may be acceptable as evidence of participation in CME activities. If you are not licensed in the US, please complete the questions online, print the AMA PRA CME credit certificate, and present it to your national medical association for review.

## Post-Test Questions

### Study Title: Australian Community Pharmacists’ Experience of Implementing a Chronic Kidney Disease Risk Assessment Service

#### CME Questions

1. Which one of the following statements regarding the epidemiology and management of chronic kidney disease (CKD) in Australia is most accurate?
  - Abnormal kidney function is estimated to affect up to 10% of primary care patients
  - The early management of CKD does not significantly affect the risk for subsequent cardiovascular disease
  - Routine screening for CKD is part of a nationwide program based in primary care offices
  - Less than 10% of patients with impaired renal function had urinary albumin levels measured in the last year
2. Which one of the following results regarding the contextual fit of CKD risk assessment in interviews of pharmacists in the current study is most accurate?
  - The risk assessment instrument was unnecessarily complex
  - The online risk assessment calculator was user-friendly
  - Customers who completed the risk assessment appreciated the service
  - Staff members were not allowed to participate in the screening process
3. Which one of the following results regarding the perceived scope of pharmacy practice in interviews of pharmacists in the current study is most accurate?
  - The best element of the risk screening program was increased interaction with general practitioners (GPs)
  - 80% of pharmacists received feedback from GPs after the screening process
  - Customers were nearly universally enthusiastic about the CKD risk screening role among pharmacists
  - Integrating CKD risk screening with other pharmacy services appeared to improve customer uptake
4. What was another major theme of interviews of pharmacists charged with screening for the risk for CKD in the current study?
  - Customers were highly engaged in CKD risk assessment
  - Pharmacists did not feel that the health risk for CKD was important
  - Customers had limited understanding of CKD and kidney function
  - Remuneration was not an issue in CKD risk assessment

## References

[1] Mills KT , Xu Y , Zhang W , Bundy JD , Chen CS , Kelly TN , A systematic analysis of worldwide population-based data on the global burden of chronic kidney disease in 2010. Kidney Int 2015;88(5):950–7. 10.1038/ki.2015.230 26221752 PMC4653075

[2] Registry ANZDATA . 39th Annual Report. Incidence of end stage kidney disease. Australia and New Zealand Dialysis and Transplant Registry, Adelaide, Australia. 2016. Accessed March 21, 2017. http://www.anzdata.org.au/.

[3] Cass A , Chadban S , Craig J , Howard H , McDonald S , Salkeld G , The economic impact of end-stage kidney disease in Australia. 2006. Accessed May 28, 2014. http://www.kidney.org.au/.

[4] Razavian M , Heeley EL , Perkovic V , Zoungas S , Weekes A , Patel AA , Cardiovascular risk management in chronic kidney disease in general practice (the AusHEART study). Nephrol Dial Transplant 2012;27(4):1396–402. 10.1093/ndt/gfr599 22053091

[5] Johnson DW . Evidence-based guide to slowing the progression of early renal insufficiency. Intern Med J 2004;34(1-2):50–7. 10.1111/j.1444-0903.2004.t01-6-.x 14748914

[6] Jose MD , Otahal P , Kirkland G , Blizzard L . Chronic kidney disease in Tasmania. Nephrology (Carlton) 2009;14(8):743–9. 10.1111/j.1440-1797.2009.01198.x 20025683

[7] Lopez-Vargas PA , Tong A , Sureshkumar P , Johnson DW , Craig JC . Prevention, detection and management of early chronic kidney disease: a systematic review of clinical practice guidelines. Nephrology (Carlton) 2013;18(9):592–604. 10.1111/nep.12119 23815515

[8] Gheewala PA , Zaidi STR , Jose MD , Bereznicki L , Peterson GM , Castelino RL . Effectiveness of targeted screening for chronic kidney disease in the community setting: a systematic review. J Nephrol 2018;31(1):27–36. 10.1007/s40620-017-0375-0 28181150

[9] Mathew TH , Corso O , Ludlow M , Boyle A , Cass A , Chadban SJ , Screening for chronic kidney disease in Australia: a pilot study in the community and workplace. Kidney Int Suppl 2010;77(Suppl 116):S9–16. 10.1038/ki.2009.538 20186177

[10] Mathew T , Corso O . Review article: early detection of chronic kidney disease in Australia: which way to go? Nephrology (Carlton) 2009;14(4):367–73. 10.1111/j.1440-1797.2009.01113.x 19563377

[11] Charting a comprehensive approach to tackling kidney disease. Pre-budget submission 2016-2017 Federal budget. Kidney Health Australia. 2017. http://kidney.org.au/. Accessed May 30, 2017.

[12] Eades CE , Ferguson JS , O’Carroll RE . Public health in community pharmacy: a systematic review of pharmacist and consumer views. BMC Public Health 2011;11(1):582. 10.1186/1471-2458-11-582 21777456 PMC3146877

[13] Benrimoj SI , Roberts AS . Providing patient care in community pharmacies in Australia. Ann Pharmacother 2005;39(11):1911–7. 10.1345/aph.1G165 16219897

[14] Thoopputra T , Pongmesa T , Newby DA , Schneider J , Li SC . Opportunistic risk screening for type 2 diabetes: exploring of application of diabetes risk assessment tool in community pharmacy in Australia and Thailand. Value Health Reg Issues 2016;9:1–7. 10.1016/j.vhri.2015.03.022 27881250

[15] Yuksel N , Majumdar SR , Biggs C , Tsuyuki RT . Community pharmacist-initiated screening program for osteoporosis: randomized controlled trial. Osteoporos Int 2010;21(3):391–8. 10.1007/s00198-009-0977-z 19499272

[16] Peterson GM , Fitzmaurice KD , Kruup H , Jackson SL , Rasiah RL . Cardiovascular risk screening program in Australian community pharmacies. Pharm World Sci 2010;32(3):373–80. 10.1007/s11096-010-9379-8 20217476

[17] Lowres N , Neubeck L , Salkeld G , Krass I , McLachlan AJ , Redfern J , Feasibility and cost-effectiveness of stroke prevention through community screening for atrial fibrillation using iPhone ECG in pharmacies. Thromb Haemost 2014;111(6):1167–76. 10.1160/TH14-03-0231 24687081

[18] Hippisley-Cox J , Coupland C . Predicting the risk of chronic kidney disease in men and women in England and Wales: prospective derivation and external validation of the QKidney Scores. BMC Fam Pract 2010;11(1):49. 10.1186/1471-2296-11-49 20565929 PMC2905345

[19] QKidney®-2016 risk calculator. ClinRisk Ltd, United Kingdom. 2010–2016. http://www.qkidney.org/index.php. Accessed April 15, 2017.

[20] Gheewala PA , Peterson GM , Zaidi STR , Jose MD , Castelino RL . Evaluation of a chronic kidney disease risk assessment service in community pharmacies. Nephrology (Carlton) 2018;10.1111/nep.13247. 29493051 10.1111/nep.13247

[21] Gheewala PA , Peterson GM , Zaidi ST , Bereznicki L , Jose MD , Castelino RL . A web-based training program to support chronic kidney disease screening by community pharmacists. Int J Clin Pharm 2016;38(5):1080–6. 10.1007/s11096-016-0330-5 27329381

[22] Palinkas LA , Horwitz SM , Green CA , Wisdom JP , Duan N , Hoagwood K . Purposeful sampling for qualitative data collection and analysis in mixed method implementation research. Adm Policy Ment Health 2015;42(5):533–44. 10.1007/s10488-013-0528-y 24193818 PMC4012002

[23] Hsieh HF , Shannon SE . Three approaches to qualitative content analysis. Qual Health Res 2005;15(9):1277–88. 10.1177/1049732305276687 16204405

[24] Thomas DR . A general inductive approach for analyzing qualitative evaluation data. Am J Eval 2006;27(2):237–46. 10.1177/1098214005283748

[25] Tong A , Sainsbury P , Craig J . Consolidated criteria for reporting qualitative research (COREQ): a 32-item checklist for interviews and focus groups. Int J Qual Health Care 2007;19(6):349–57. 10.1093/intqhc/mzm042 17872937

[26] Van C , Krass I , Mitchell B . General practitioner perceptions of extended pharmacy services and modes of collaboration with pharmacists. Journal of Pharmacy Practice and Research. 2007;37(3):182–6. 10.1002/j.2055-2335.2007.tb00739.x

[27] McMillan SS , Kelly F , Sav A , King MA , Whitty JA , Wheeler AJ . Consumer and carer views of Australian community pharmacy practice: awareness, experiences and expectations. J Pharm Health Serv Res 2014;5(1):29–36. 10.1111/jphs.12043

[28] Gidman W , Cowley J . A qualitative exploration of opinions on the community pharmacists’ role amongst the general public in Scotland. Int J Pharm Pract 2013;21(5):288–96. 10.1111/ijpp.12008 23418884

[29] Lowres N , Krass I , Neubeck L , Redfern J , McLachlan AJ , Bennett AA , Atrial fibrillation screening in pharmacies using an iPhone ECG: a qualitative review of implementation. Int J Clin Pharm 2015;37(6):1111–20. 10.1007/s11096-015-0169-1 26202627

[30] Cheong AT , Khoo EM , Tong SF , Liew SM . To check or not to check? A qualitative study on how the public decides on health checks for cardiovascular disease prevention. PLoS One 2016;11(7):e0159438. Erratum in: PLoS One 11 (8): e0162152. 10.1371/journal.pone.0159438 27415432 PMC4945067

[31] Guest G , Bunce A , Johnson L . How many interviews are enough? Field Methods 2006;18(1):59–82. 10.1177/1525822X05279903

